# Genomic Access to Monarch Migration Using TALEN and CRISPR/Cas9-Mediated Targeted Mutagenesis

**DOI:** 10.1534/g3.116.027029

**Published:** 2016-02-01

**Authors:** Matthew J. Markert, Ying Zhang, Metewo S. Enuameh, Steven M. Reppert, Scot A. Wolfe, Christine Merlin

**Affiliations:** * Department of Biology and Center for Biological Clocks Research, Texas A&M University, College Station, Texas 77843; † Department of Biochemistry and Molecular Pharmacology, University of Massachusetts Medical School, Worcester, Massachusetts 01605; ‡ Department of Neurobiology, University of Massachusetts Medical School, Worcester, Massachusetts 01605

**Keywords:** TALENs, CRISPR, insect, germline targeting, clock genes

## Abstract

The eastern North American monarch butterfly, *Danaus plexippus*, is an emerging model system to study the neural, molecular, and genetic basis of animal long-distance migration and animal clockwork mechanisms. While genomic studies have provided new insight into migration-associated and circadian clock genes, the general lack of simple and versatile reverse-genetic methods has limited *in vivo* functional analysis of candidate genes in this species. Here, we report the establishment of highly efficient and heritable gene mutagenesis methods in the monarch butterfly using transcriptional activator-like effector nucleases (TALENs) and CRISPR-associated RNA-guided nuclease Cas9 (CRISPR/Cas9). Using two clock gene loci, *cryptochrome 2* and *clock* (*clk*), as candidates, we show that both TALENs and CRISPR/Cas9 generate high-frequency nonhomologous end-joining (NHEJ)-mediated mutations at targeted sites (up to 100%), and that injecting fewer than 100 eggs is sufficient to recover mutant progeny and generate monarch knockout lines in about 3 months. Our study also genetically defines monarch CLK as an essential component of the transcriptional activation complex of the circadian clock. The methods presented should not only greatly accelerate functional analyses of many aspects of monarch biology, but are also anticipated to facilitate the development of these tools in other nontraditional insect species as well as the development of homology-directed knock-ins.

With their rich biology and extraordinary diversity, butterflies and moths alike offer great opportunities to study the molecular and genetic basis of fascinating biological phenomena not manifested by conventional genetic model organisms. A striking example is the seasonal monarch butterfly’s (*Danaus plexippus*) long-distance migration from its northern breeding range in southeastern Canada and northeastern USA to its overwintering roosting sites in central Mexico (Urquhart 1987). Despite its spectacular nature and an increasing public interest in its conservation, we still know very little about the genetic basis governing this behavior. With the availability of a high-quality reference genome (Zhan *et al.* 2011), the monarch represents a unique model to decipher the genetic and epigenetic basis underlying animal long-distance migration and the associated timing mechanisms (Reppert *et al.* 2016).

Northeastern American migratory monarch populations possess several generations of migratory and nonmigratory forms, all equipped with the same core genetic makeup. In the fall, the migratory state is initiated in response to changing environmental factors predicting winter’s arrival (*i.e.*, decreasing day length and temperature; Reppert *et al.* 2010). Migrants exhibit an oriented flight guided by a sun compass that is time-compensated by circadian clocks in the antennae, allowing them to maintain a fixed flight direction throughout the day (Perez *et al.* 1997; Mouritsen and Frost 2002; Froy *et al.* 2003; Merlin *et al.* 2009; Guerra *et al.* 2012). While a recent population genomic analysis of 101 *Danaus* genome sequences from migratory and nonmigratory monarch populations identified a set of over 500 candidate genes associated with migration (Zhan *et al.* 2014), understanding the role of these genes in the biology of monarch migration will require further functional genomic approaches, such as efficient and accessible reverse genetic tools to dissect gene function *in vivo*.

Engineered nucleases have proved to be powerful for genome editing in a wide range of model and nonmodel organisms (Gaj *et al.* 2013; Carroll 2014). By inducing double-stranded breaks (DSBs) at specific genomic sites, they favor the introduction of small random insertions/deletions (indels) through imperfect nonhomologous-end joining repair, thereby allowing the generation of gene knockouts. In fact, zinc-finger nucleases have previously enabled heritable targeted mutagenesis *in vivo* of a monarch clock gene, *cryptochrome 2* (*cry2*), which encodes the main transcriptional repressor of the CLOCK (CLK):CYCLE (CYC) activation complex within the migration-relevant clock mechanism (Merlin *et al.* 2013). Although the zinc-finger nucleases approach provided a proof-of-principle for the use of engineered endonucleases in butterflies and established reliable methods for nuclease delivery and mutation screening approaches, its efficiency failed to reach a standard enabling facile and rapid recovery of germline mutants. Notably, the recovery of a few germline mutants required the injection of thousands of eggs (Merlin *et al.* 2013). Other engineered nucleases, including transcription activator-like effector nucleases (TALENs) and RNA-guided nucleases derived from clustered regularly interspaced short palindromic repeats (CRISPR)/Cas9 nuclease, have enabled efficient heritable targeted gene disruption in another lepidopteran, the silkworm *Bombyx mori* (Ma *et al.* 2012; Sajwan *et al.* 2013; Wang *et al.* 2013; Liu *et al.* 2014; Wei *et al.* 2014). CRISPR/Cas9 has also been recently reported to induce somatic mutations with high efficiencies in the swallowtail butterfly (Li *et al.* 2015). However, efficient germline transmission of Cas9-mediated mutations in a butterfly has yet to be reported.

Here, we report the generation of highly efficient, heritable gene knockouts at two clock gene loci, *cry2* and *clk*, in the monarch butterfly using TALENs and CRISPR/Cas9. We demonstrate that, for both target genes and nuclease platforms, injecting less than 100 eggs is sufficient to recover mutant progeny and generate monarch knockout lines in about 3 months. We also present the functional characterization of a monarch *clk* knockout, thereby establishing a novel reagent to study the role of circadian clocks in monarch migration. Our data provide new technological resources to rapidly establish genetic mutant lines in the monarch butterfly that will ultimately accelerate the quest to elucidate the genetic basis of their remarkable migration abilities.

## Materials and Methods

### Monarch butterfly husbandry

Monarch butterflies were reared in the laboratory as previously described (Merlin *et al.* 2013). Gravid females were placed in cages to lay eggs on potted tropical milkweed plants (*Asclepias curassavica*). Eggs were transferred to petri dishes containing milkweed leaves until hatched. Larvae were fed milkweed up to their second instar and then transferred onto individual cups of semiartificial diet. Larvae were raised in Percival incubators at 25°, 70% humidity, and under 15 hr light, 9 hr dark (LD, 15:9) conditions. Adult monarchs were housed in glassine envelopes in the same lighting and temperature conditions, and were manually fed a 25% honey solution daily.

### TALEN target selection and assembly

TALEN target sites were selected within the 5′-end of the *cry2* and *clk* coding sequences. The *cry2* TALEN target site was chosen to overlap with the previously described *cry2* ZFN target site (Merlin *et al.* 2013). Criteria for target site selection included the presence of an endogenous restriction endonuclease site for the assessment of TALEN activity and genotyping of carriers of null alleles, and a 5′-T within each monomer half-site. The standard Repeat Variable Di-residues (*i.e.*, Ade = NI; Cyt = HD; Gua = NN; Thy = NG) were utilized for base recognition in the remaining sequence positions. The TALE monomers comprised 16.5–19.5 modules with a 16 bp gap separating the TALEN target sites. TALE module arrays were constructed using a modified Voytas golden gate system (Cermak *et al.* 2011), in which the TALE arrays were assembled into modified pJDS vectors containing the wild-type FokI nuclease domain (Sander *et al.* 2011), as described previously (Gupta *et al.* 2013).

### gRNA design and construction

The gRNA target sites were selected within the 5′-end of the *cry2* and *clk* coding sequences using the Jack Lin’s CRISPR/Cas9 gRNA finder (http://spot.colorado.edu/~slin/cas9.html). The *cry2* gRNA target site was chosen to overlap with the previously described *cry2* ZFN target site (Merlin *et al.* 2013) and the TALEN target site. The gRNA expression vectors were constructed by inserting annealed synthetic oligomers into the DR274 plasmid from Addgene (Hwang *et al.* 2013) at the *Bsa*I cleavage sites.

### Synthesis of TALEN mRNA, Cas9 mRNA, and sgRNA

*In vitro* transcription of TALEN mRNAs was performed using the mMessage mMachine T7 kit (Ambion) using pJDS-TALEN constructs linearized with *Pme*I and phenol-chloroform purified, and were polyadenylated using the Poly(A) Tailing Kit (Ambion), according to the manufacturer’s instructions. *In vitro* transcription of *Streptococcus pyogenes* Cas9 mRNA was performed using the mMessage mMachine T3 transcription kit (Ambion), using the pCS2-nCas9n expression plasmid from Addgene (Jao *et al.* 2013) linearized with *Xba*I and purified with phenol-chloroform. The resulting capped PolyA mRNAs were purified by acid-phenol-chloroform extraction and resuspended in RNase-free water following isopropanol precipitation. sgRNAs were *in vitro* transcribed from sgRNA-containing DR274 vectors linearized with *Dra*I and purified by phenol-chloroform extraction, using T7 RNA polymerase (Promega) according to the manufacturer’s instructions. RNAs were quantitated by spectrophotometry (NanoDrop 1000) and diluted in RNase-free water to a final concentration of 0.5 μg/μl for *cry2* TALENs and *clk* TALENs, 0.5 μg/μl for Cas9, and of 0.1 μg/μl for *cry2* and *clk* sgRNAs.

### Egg microinjection

To perform microinjections, eggs were collected as previously described (Merlin *et al.* 2013) and injected within 20 min of being laid. A solution containing TALEN mRNAs or Cas9 mRNA, sgRNAs, and blue food coloring was loaded into a pulled borosilicate glass needle (World Precision Instruments, Inc.) attached to an IM 300 microinjector (Narishige), and injections were performed under a dissecting microscope. After injection, embryos were placed in an incubator at 25° and 70% relative humidity. Developing embryos were transferred into individual small petri dishes until larvae hatched. Larvae were fed milkweed leaves until the second larval instar before being transferred onto a semiartificial diet.

### Analysis of TALEN-induced mutations using restriction assays and mutant line establishment

Surviving fourth instar larvae were screened for the presence of mutations at the targeted site by noninvasive genotyping as previously described (Merlin *et al.* 2013). Genomic DNA from larval sensors was extracted using 0.01 × proteinase K (Sigma) in lysis buffer (100 mM Tris pH 8.0, 25 mM NaCl, 1 mM EDTA). Fragments flanking the targeted regions were amplified by PCR using the primers in Supplemental Material, Table S1, and subjected to restriction fragment length polymorphism analysis using *Eag*I for *cry2* or *Ngo*MIV for *clk*. After resolution of the digestion reaction using agarose gel electrophoresis and visualization with EtBr staining, the frequency of NHEJ-mediated indels was estimated for each founder by quantification of ethidium bromide staining and densitometry of the resistant band relative to wild-type fragments using ImageJ 1.49. Fragments corresponding to mutated alleles were gel purified, subcloned, and sequenced using the same primers. Larvae presenting a high degree of targeting in somatic cells were selected for further analysis and reared to adulthood. Adults were hand-paired in individual cages either with virgin wild-type or mosaic individuals of the opposite sex to establish lines. Eggs were collected from each line, and the hatched larvae were raised and screened for the presence of mutated alleles as described above.

### Analysis of CRISPR/Cas9-induced mutations using a Cas9 in vitro cleavage assay

For *in vitro* cleavage assay, recombinant Cas9 protein was produced in *Escherichia coli* using a pET28-Cas9 expression plasmid purchased from PNA Bio Inc. The recombinant Cas9 protein was expressed in BL21 (DE3) competent cells induced with IPTG and purified, as previously described (Kim *et al.* 2014). Cas9 protein was purified over a Nickel column using Ni-NTA His-Bind Resin (Novagen) and analyzed by SDS-PAGE.

For analysis of CRISPR/Cas9 induced mutations, PCR fragments flanking the targeted regions were amplified with *cry2* and *clk* primers (given in Table S1). PCR products were purified using 1.5 × modified Sera-Mag Magnetic Speed-beads (Fisher) as described by Rohland and Reich (2012) and resuspended in 10 μl of water. The Cas9-based cleavage assay was performed as previously described (Kim *et al.* 2014), with slight modifications. Purified PCR products (150–200 ng) were incubated for 3 hr at 37° with purified Cas9 protein (100 ng), sgRNA (100–300 ng) used for targeting, BSA (1 μg/μl; New England Biolabs, NEB), and NEB Buffer 3 (1X). After incubation, 4 μg of RNase A (Amresco) was added and reactions were incubated for an additional 2 hr at 37°. Reactions were stopped using a 6 × stop solution described in (Kim *et al.* 2014). Products were purified using 1.5 × modified Sera-Mag Magnetic Speed-beads before being resolved with agarose gel electrophoresis and EtBr staining. The frequency of NHEJ-mediated indels was estimated as described for TALENs, and fragments corresponding to mutated alleles were gel purified and sequenced using the same primers used to generate the PCR amplicons. To assess germline targeting rates, larvae were selected and their progeny recovered as described above.

### Eclosion behavior

Eclosion behavior was performed as previously described with slight modifications (Merlin *et al.* 2013). Monarch larvae were housed in LD 15:9 in a Percival incubator at 25° and 70% humidity through pupation, and pupae were transferred to constant darkness (DD) 1 or 2 d prior to the predicted time of adult eclosion. Eclosion behavior was monitored continuously over time using a digital video recorder and night vision security cameras (Night Owl Security L-45-4511) mounted inside the incubator. Eclosion data were analyzed and plotted as 1 hr bins.

### Real-time qPCR

Brains from adult monarch butterflies entrained to seven LD cycles were dissected under red light on the first day of transfer into DD. Dissections were performed in 0.5 × RNA later (Ambion) to avoid RNA degradation, and brains free of eye photoreceptors were stored at –80° until use. Total RNA was extracted using an RNeasy Mini kit (Qiagen), treated with RQ1 DNase (Promega), and random hexamers (Promega) were used to prime reverse transcription with Superscript II Reverse Transcriptase (Invitrogen), all according to the manufacturers’ instructions. Quantifications of clock gene expression were done using SYBR Green-based real-time quantitative PCR assays with an ABI 7500 Fast Real-Time PCR system (Applied Biosystems). PCR reactions were assembled by combining two master mixes: the first mix contained 5 μl of iTaq Universal SYBR Green Supermix (Bio-Rad) and forward and reverse primers (5 picomole each) per reaction, and the second mix contained approximately 75 ng of cDNA template and the water needed to bring each reaction to a final volume of 10 μl. Each reaction was assembled by consecutively aliquoting each mix into a PCR plate. The monarch *per*, *tim*, *clock*, and control *rp49* primers were as follows (F, forward primer; R, reverse primer): monarch *perF*, 5’-AGTGAAGCGTCCCTCAAAACA-3’; *perR*, 5’-TGGCGACGAGCATCTGTGT-3’; monarch *timF*, 5’-GGACGGCAGCGGATACG-3’; *timR*, 5’-CGCCGTTTCGCACACA-3’; monarch *rp49F*, 5’-TGC GCAGGCGTTTTAAGG-3’; *rp49R*, 5’-TTGTTTGATCCGTAACCAATGC-3’; monarch *clockF*, 5’-CATACCGGTTATAGTCGCACAAGA-3’; *clockR*, 5’-GATGGGTTCGCTGCAACTG-3’. The near 100% efficiency of each primer set was validated by determining the slope of Ct *vs.* dilution plot on a 3 × 10^4^ dilution series. Individual reactions were used to quantify each RNA level in a given cDNA sample, and the average Ct from duplicated reactions within the same run was used for quantification. The data for each gene in a given condition (genotype and time of day) were normalized to *rp49* as an internal control, and normalized to the mean of one time point within a set for statistics.

### Statistical analysis

*P*-values were calculated using a Student’s *t*-test and one-way or two-way ANOVAs using the QI Macros Statistical software for Excel.

### Data availability

The monarch *clk* knockout line is available upon request.

## Results

### Knocking out monarch clock genes using TALENs

To assess the capacity of TALENs to efficiently produce loss-of-function mutations in the monarch butterfly, we chose to target two clock genes: *cryptochrome 2* (*cry2*), which encodes the main transcriptional repressor of the monarch circadian clock (Zhu *et al.* 2008; Merlin *et al.* 2013), and *clock* (*clk*), which encodes a component of the heterodimeric transcriptional activator complex of the clock (Zhu *et al.* 2008).

**Figure 1:**
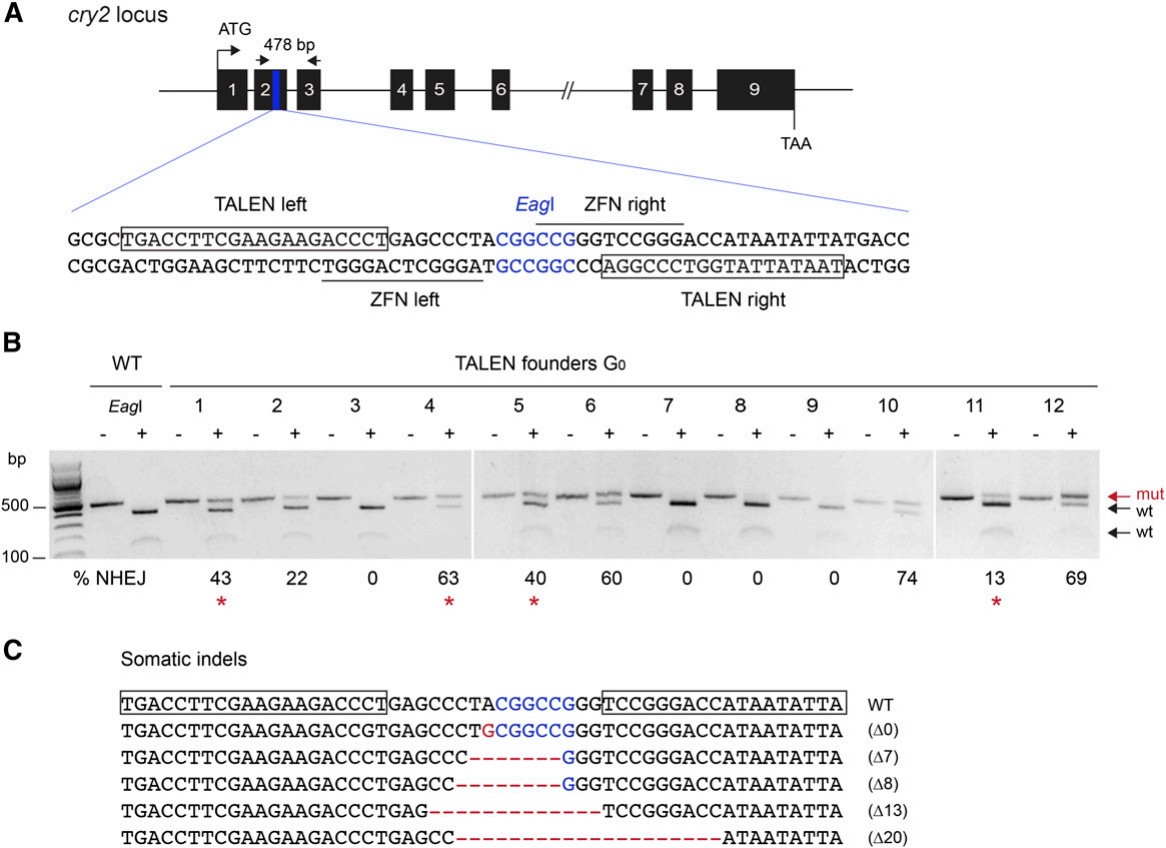
Targeted mutagenesis induced by microinjection of *cry2* TALEN mRNAs into monarch butterfly embryos. (A) Schematic diagram of the TALEN targeting sites. The black line depicts the genomic locus of monarch *cry2*, and the numbered black boxes represent the 9 exons of *cry2*. The blue region in exon 2 is expanded to provide the sequence that includes the TALEN binding sites (shown in boxes) and the previously described ZFN binding sites (underlined, Merlin *et al.* 2013). The restriction site (*Eag*I) in the spacer is shown in blue. Arrows on top represent the position of the primers used to amplify the 478 bp targeted region for analysis of mutagenic lesions in B (*cry2* F1, *cry2* R1 in Table S1). (B) Targeted mutagenesis in representative founder G_0_ butterflies validated by PCR and *Eag*I digestion. For each founder, the PCR product was either not subjected (–) or subjected to *Eag*I (+). Genomic amplicons carrying nonhomologous end-joining (NHEJ)-induced mutations (mut; red arrow) leading to the loss of the *Eag*I site are resistant to restriction enzyme digestion. The frequency of NHEJ-mediated indels provided under each founder was estimated by relative quantification of the resistant and digested products. Red stars, founders crossed to determine germline targeting rates. (C) TALEN-induced mutations in somatic cells of larvae surviving the injection. TALEN binding sites are boxed on the wild-type sequence. *Eag*I site is in blue, and the positions of deletion and insertions are in red (dashes and letters, respectively). TALEN, transcriptional activator-like effector nucleases; ZFN, zinc-finger nuclease.

The *cry2* locus was selected as an ideal candidate for direct comparison of targeting efficiencies between different nuclease-mediated targeting technologies, including ZFNs (Merlin *et al.* 2013). The TALE binding sites were therefore chosen to overlap with the binding sites of the ZFN pair previously used to target the second exon of *cry2* (Figure 1A). To test the efficiency of TALEN-mediated targeted mutagenesis at the *cry2* locus, embryos in early stages of development (*i.e.*, 20 min postoviposition) were injected with 0.5 μg/μl of mRNAs encoding the TALEN pair. Given that fertilization occurs at oviposition and the zygote nucleus is formed and starts dividing soon after, injecting shortly after the eggs have been laid likely increases the chances to target the nuclei that will later give rise to germline precursors (Merlin *et al.* 2013). TALEN-induced mutations (insertions or deletions; indels) were screened in surviving larvae using polymerase chain reaction (PCR)-based assays from clipped cuticular expansions, as previously described (Merlin *et al.* 2013).

To facilitate the screening of indels at the targeted site and readily assess the level of mosaicism in potential founders, the spacer between the TALE binding sites was chosen to contain an endogenous restriction site, *Eag*I (Figure 1A). PCR amplicons carrying indels that disrupt the enzyme recognition site become resistant to digestion, and the percentage or rate of NHEJ-mediated lesions can be estimated in somatic tissues by quantifying the proportion of uncleaved mutated fragments relative to cleaved wild-type ones (Figure 1B). Complete digestion of the PCR-amplified fragments from noninjected wild-type larvae was observed (Figure 1B). Digestion-resistant fragments were found in somatic tissue of ∼65% of the larvae surviving injections (11/17) from a pool of 86 injected embryos. In these targeted individuals, the rates of mutagenic lesions were estimated to range from 5–74% (Figure 1B, Figure S1A, and Table 1). This stands in striking contrast to the efficiency of ZFN-mediated targeted mutagenesis previously reported at this locus, with only four somatic mutants recovered out of ∼1200 injected eggs, albeit with comparable mosaicism levels (Table 1, Merlin *et al.* 2013). Cloning and sequencing of the purified resistant PCR fragments from one individual confirmed the presence of frameshifting microdeletions at the targeted site (Figure 1C), similar to what has been previously described with ZFNs (Merlin *et al.* 2013).

**Table 1:** ZFN-, TALEN-, and CRISPR/Cas9-induced mutagenesis after mRNA microinjection in the monarch butterfly

ZFNs/TALENs/CRISPRs	Number of Injected Eggs	Hatching Rate, %	Live Larvae, *n* (*%*)	Somatic Mutants, *n* (*%*)	Germline Mutation Rate, % (Mut crossed, *n*)	Reference
*cry2* ZFNs	1194	4.8	45 (3.8)	4 (8.9)	44.5 (2)	Merlin *et al.* 2013
*cry2* TALENs	86	22	17 (19.8)	11 (64.7)	4.1 (4)	This study
*cry2* CRISPR (2 sgRNAs)	88	21.6	13 (14.8)	8 (61.5)	100 (2)	This study
*clock* TALENs	90	21.1	17 (18.9)	17 (100)	52.4 (4)	This study
*clock* CRISPR (1 sgRNA)	93	39.8	19 (20.4)	11 (57.9)	12.2 (2)	This study

The percentage of live larvae corresponds to the number of larvae that survived to adulthood relative to the number of injected eggs. The percentage of somatic mutants corresponds to the number of live larvae that presented any degree of somatic mosaicism. The germline mutation rate corresponds to the percentage of progeny carrying a mutated allele from the total of progeny tested. ZFNs, zinc-finger nucleases; TALENs, transcriptional activator-like effector nucleases; CRISPR, clustered regularly interspaced short palindromic repeats.

To determine whether TALEN-induced mutations could be transmitted through the monarch germline, we intercrossed two adult mosaic males to two adult mosaic females presenting the highest levels of mosaicism in somatic cells (two mating pairs; Figure 1B, red stars). From the 24 F_1_ larvae that eclosed out of the 73 oviposited eggs, one carried a mutated *cry2* allele (∼4%; Table 1).

To expand our analysis and demonstrate that TALENs can be used efficiently to target other loci within the monarch genome, we next applied this technology to the *clk* locus. The choice of this target was motivated by the fact that a loss-of-function monarch *clk* knockout would constitute a complementary reagent to the already existing monarch *cry2* knockout, for future functional studies on the circadian control of the monarch butterfly seasonal long-distance migration. Indeed, monarch CLK has been shown *in vitro* to function in a manner similar to its *Drosophila* homolog as a transcriptional activator within the CLK:CYC heterodimeric complex, therefore having an opposite role to CRY2 (Zhu *et al.* 2008; Allada *et al.* 1998).

**Figure 2:**
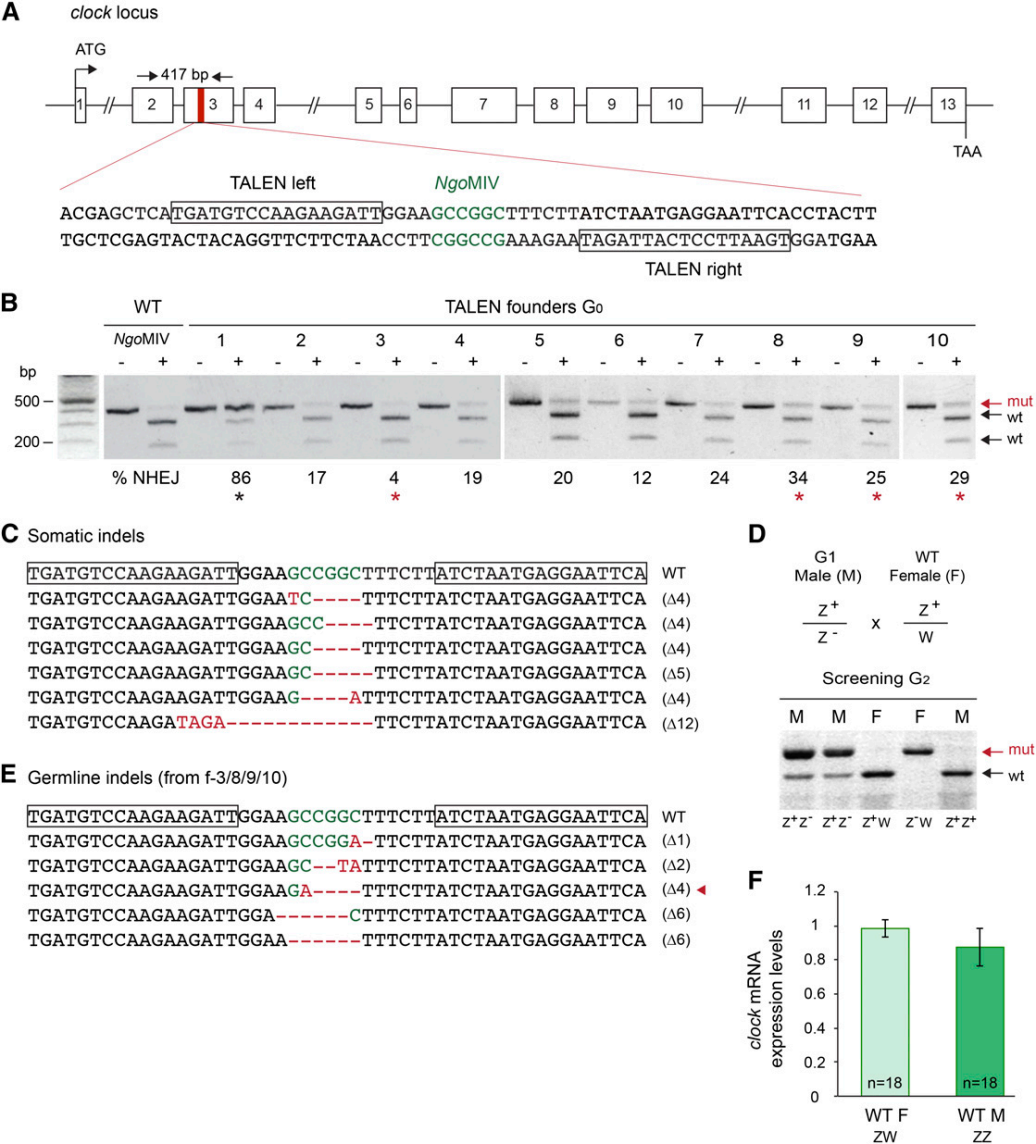
TALEN-mediated targeted mutagenesis in somatic and germline cells of monarch butterfly *clock*. (A) Schematic diagram of the monarch *clock* genomic locus and the TALEN targeting sites. The red region in exon 3 is expanded to provide the sequence that includes the TALEN binding sites (shown in boxes). The restriction site (*Ngo*MIV) in the spacer is shown in green. Arrows on top represent the position of the primers used to amplify the 417 bp targeted region for analysis of mutagenic lesions in B (*clock* F1, *clock* R1 in Table S1). (B) Targeted mutagenesis in representative founder G_0_ butterflies validated by PCR and *Ngo*MIV digestion. For each founder, the PCR product was either not subjected (–) or subjected (+) to *Ngo*MIV. Genomic amplicons carrying NHEJ-induced mutations (mut; red arrow) leading to the loss of the *Ngo*MIV site are resistant to restriction enzyme digestion. Estimation of the frequency of NHEJ-mediated indels is provided under each founder. Red stars, founders crossed to determine germline targeting rates. Black star, highly targeted G_0_ butterfly that did not survive to adulthood. (C) TALEN-induced mutations in somatic cells of larvae surviving the injection. TALEN binding sites are boxed on the wild-type sequence. *Ngo*MIV site is in green, and the positions of deletions and insertions are in red (dashes and letters, respectively). (D) The *clock* gene is located on the Z chromosome. Crosses between heterozygote clock mutant (G_1_) and wild-type butterflies produce progeny that include heterozygote males (M) and homozygote females (F) for the mutated allele (mut; red arrow). (E) TALEN-induced mutations in the germline of founders 3, 8, 9, and 10. Red arrowhead, butterfly selected to establish a *clock* mutant line. (F) Relative expression level of *clock* in brains of wild-type monarch females (WT F) and males (WT M). Values are mean ± SEM of 18 animals, collected every 3 hr in triplicates over 24 hr. *t*-test: *P* = 0.37. PCR, polymerase chain reaction; SEM, standard error of the mean; TALEN, transcriptional activator-like effector nuclease; WT, wild-type monarchs.

A high quality TALEN site was identified in exon 3 of the monarch *clk* gene, with the two TALE binding sites flanking a spacer containing an endogenous restriction site, *Ngo*MIV, which can be used to detect the presence of indels and estimate targeting frequencies, as described above (Figure 2A). Frameshift mutations at this site are predicted to result in the production of truncated, nonfunctional proteins lacking the basic-helix-loop-helix-PER-ARNT-SIM domain required for the formation of the functional CLK:CYC heterodimer (Allada *et al.* 1998). To test the efficiency of TALEN-mediated targeted mutagenesis at the *clk* locus, embryos were injected with 0.5 μg/μl of mRNAs encoding the TALEN pair, and the presence of indels at the targeted site was examined in surviving larvae by restriction digest. Approximately 19% of injected embryos (17/90) survived injection, and all exhibited mosaicism at the targeted site with frequencies of mutated lesions ranging from 3–86% (Figure 2B, Figure S1B, and Table 1). Cloning and sequencing of the purified resistant PCR fragments from one individual confirmed the presence of frameshifting microdeletions at the targeted site (Figure 2C).

To determine the germline transmission rates of TALEN-induced mutations and establish a *clk* knockout line, we screened the progeny of two intercrossed mosaic males and two mosaic females, selected among the most highly targeted individuals (Figure 2B, red stars). Out of 68 F_1_ larvae, ∼52% carried at least one mutated allele, with 21 heterozygotes and 12 homozygotes. Sexing the corresponding adults revealed that all homozygous mutants were females, all heterozygous mutants were males, and wild-type butterflies were of both sexes. This suggested that *clk* is sex-linked and located on the Z sex chromosome [in lepidopterans, the female is the heterogametic (ZW) sex and the male is homogametic (ZZ); Figure 2D], adding to the list of sex-linked clock genes in Lepidoptera (Gotter *et al.* 1999; Abe *et al.* 2010). To unambiguously determine that the homozygous mutants were resulting from sex-based segregation, and not from biallelic mutations transmitted from two mosaic parents, we sequenced the resistant fragments of four female and seven male F_1_ progeny. Consistent with *clk* being linked to the Z chromosome, we found a single mutation per animal tested. All these mutations were microdeletions, and a 4 bp-del mutant that would generate a truncated, nonfunctional protein (Figure 2E; red arrowhead.) was chosen to establish a *clk* knockout line. Genetic evidence that monarch *clk* was Z-linked prompted us to test whether it would be dosage compensated between sexes, as sex chromosome dosage compensation in the lepidopteran clade is still debated (Walters *et al.* 2015). In the absence of dosage compensation, *clk* expression in females would be half the expression level in males. We found no statistical differences in *clk* expression in wild-type monarch brains between sexes, suggesting complete dosage compensation in the monarch butterfly, at least at this Z-linked locus (Figure 2F, Student’s *t*-test, *P* = 0.37; Figure S2).

### CRISPR/Cas9 mediate efficient clock gene knockouts in the monarch butterfly using single sgRNA and dual sgRNAs

With a more straightforward design and faster construction time, the bacterial-derived CRISPR/Cas9 system has emerged as an alternative to TALENs for the generation of gene knockouts. We tested its efficiency in the monarch butterfly, focusing our study on the genes previously targeted with ZFNs and TALENs, *clk* and *cry2*, for direct comparison.

**Figure 3:**
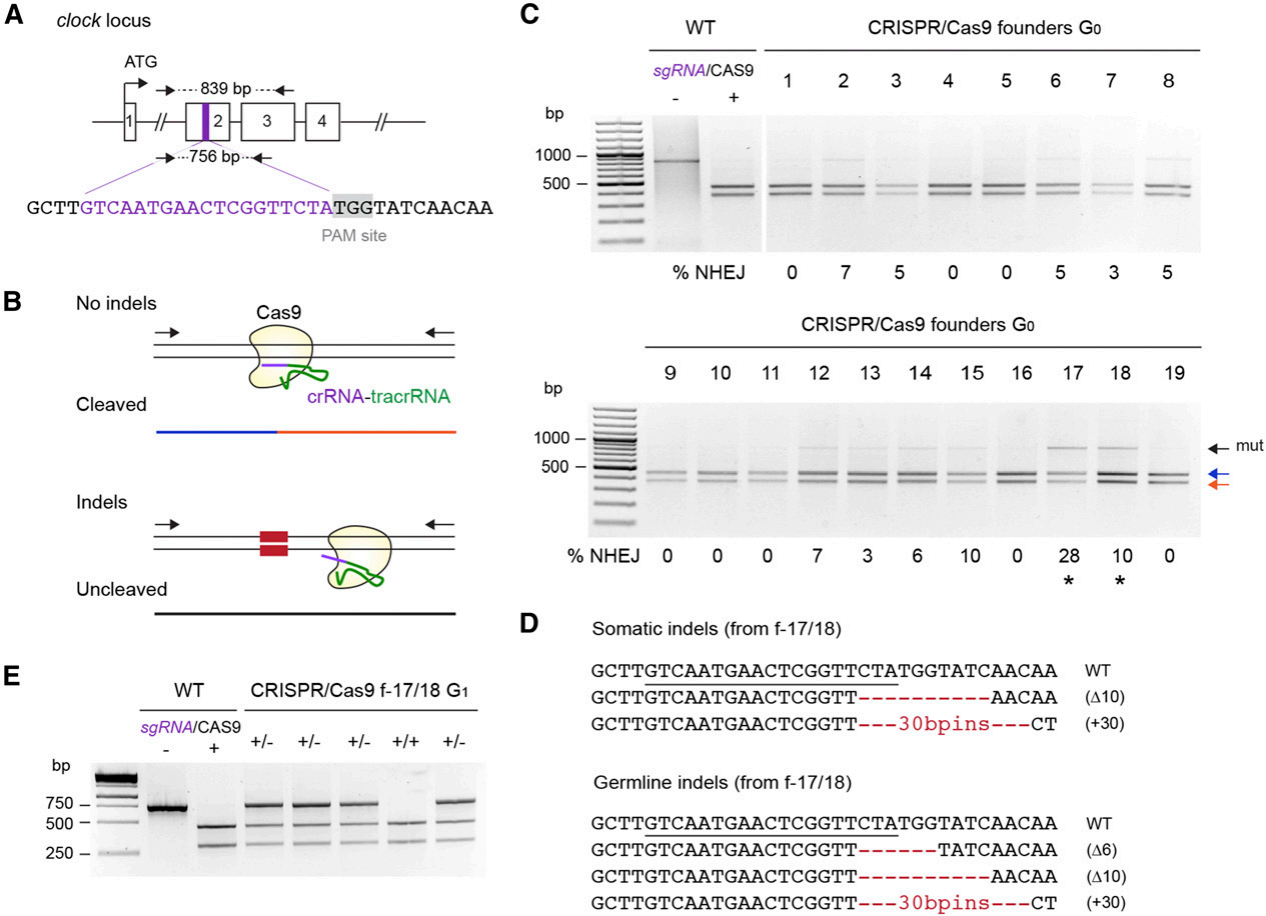
Heritable CRISPR/Cas9-mediated targeted mutagenesis of monarch butterfly *clock*. (A) Schematic of part of the monarch *clock* genomic locus containing the CRISPR/Cas9 target site. The purple region in exon 2 is expanded to provide the sequence targeted for genome editing by a single guide RNA (sgRNA, purple letters). The protospacer adjacent motif (PAM site, 5′-NGG-3′) 3′ of the target sequence is highlighted in gray. Arrows on top and bottom represent the positions of the primers used to amplify the 839 bp (*clock* F2, *clock* R1; Table S1) and 756 bp (*clock* F2, *clock* R2; Table S1) targeted regions for analysis of mutagenic lesions in C and E, respectively. (B) Diagram of the *in vitro* cleavage assay used to detect mutagenic lesions showing a PCR amplicon subjected to the sgRNA and the Cas9 protein. Blue and red lines correspond to a wild-type (WT) genomic fragment cleaved by Cas9. Fragments with mutations induced by NHEJ at the site targeted by the sgRNA (red boxes) are uncleaved (black line). (C) Detection of mutagenic lesions at the *clock* locus in somatic cells of founder G_0_ butterflies. For each founder, a PCR fragment was subjected to a Cas9-based *in vitro* cleavage assay. Blue and red arrows, WT fragments. Black arrow, amplicons carrying mutations (mut) at the site targeted by the sgRNA. Estimation of the frequency of NHEJ-mediated indels is provided under each founder. Black stars, founders selected for crosses to determine germline targeting rates. (D) CRISPR/Cas9-induced mutations in somatic and germline cells of founders 17 and 18. The sgRNA binding site is underlined on the wild-type sequence. Red dashes and red letters, deletion and insertions, respectively. (E) Genotyping of G_1_ butterflies from founders 17 and 18 backcrossed to WT using the Cas9 *in vitro* cleavage assay. Heterozygote butterflies carrying the mutated allele are robustly discriminated by the presence of an additional uncleaved PCR fragment. CRISPR, clustered regularly interspaced short palindromic repeats; NHEJ, nonhomologous end-joining; PCR, polymerase chain reaction.

For targeting of *clk*, we coinjected monarch eggs with 0.1 μg/μl of an *in vitro* transcribed single guide RNA (sgRNA) complementary to a 19 bp DNA sequence in the second exon, along with 0.5 μg/μl of mRNA encoding Cas9 (Figure 3A). To screen for CRISPR/Cas9-mediated indels in surviving larvae and quantitatively estimate their occurrence rate in somatic cells, we used a Cas9-based *in vitro* cleavage assay modified from (Kim *et al.* 2014). Unlike the T7 endonuclease I and mismatch-sensitive surveyor assays (Kim *et al.* 2009), this assay is quantitative and substantially more cost-effective than high-throughput sequencing (Kim *et al.* 2014). It relies on the sgRNA used for introducing *in vivo* mutations and a recombinant Cas9 protein to detect the presence of lesions via the loss of sensitivity of PCR fragments spanning the targeted site to Cas9. In this assay, the sgRNA hybridizes with its target sequence on wild-type fragments but fails to do so with mutated target sequences, resulting in cleavage of the wild-type sequences only. The occurrence and frequency of NHEJ-mediated indels can then be estimated by quantifying the proportion of uncleaved PCR fragments relative to the total PCR product (cleaved and uncleaved; Figure 3B). Comparable to what we found with TALENs, ∼20% of the injected embryos developed into healthy larvae (19/93; Table 1), and ∼50% of those individuals presented indels at rates ranging from 3–28% (Figure 3C and Table 1). Cloning and sequencing of the resistant PCR fragments from two potential founders confirmed the presence of indels in somatic cells (Figure 3D). Interestingly, the same mutations were recovered in the progeny of the founders backcrossed to wild-type monarchs (Figure 3, D and E), supporting the hypothesis that injecting very early into newly oviposited eggs is a robust strategy to achieve high germline transmission rates in the monarch, regardless of the genome targeting technology applied.

**Figure 4:**
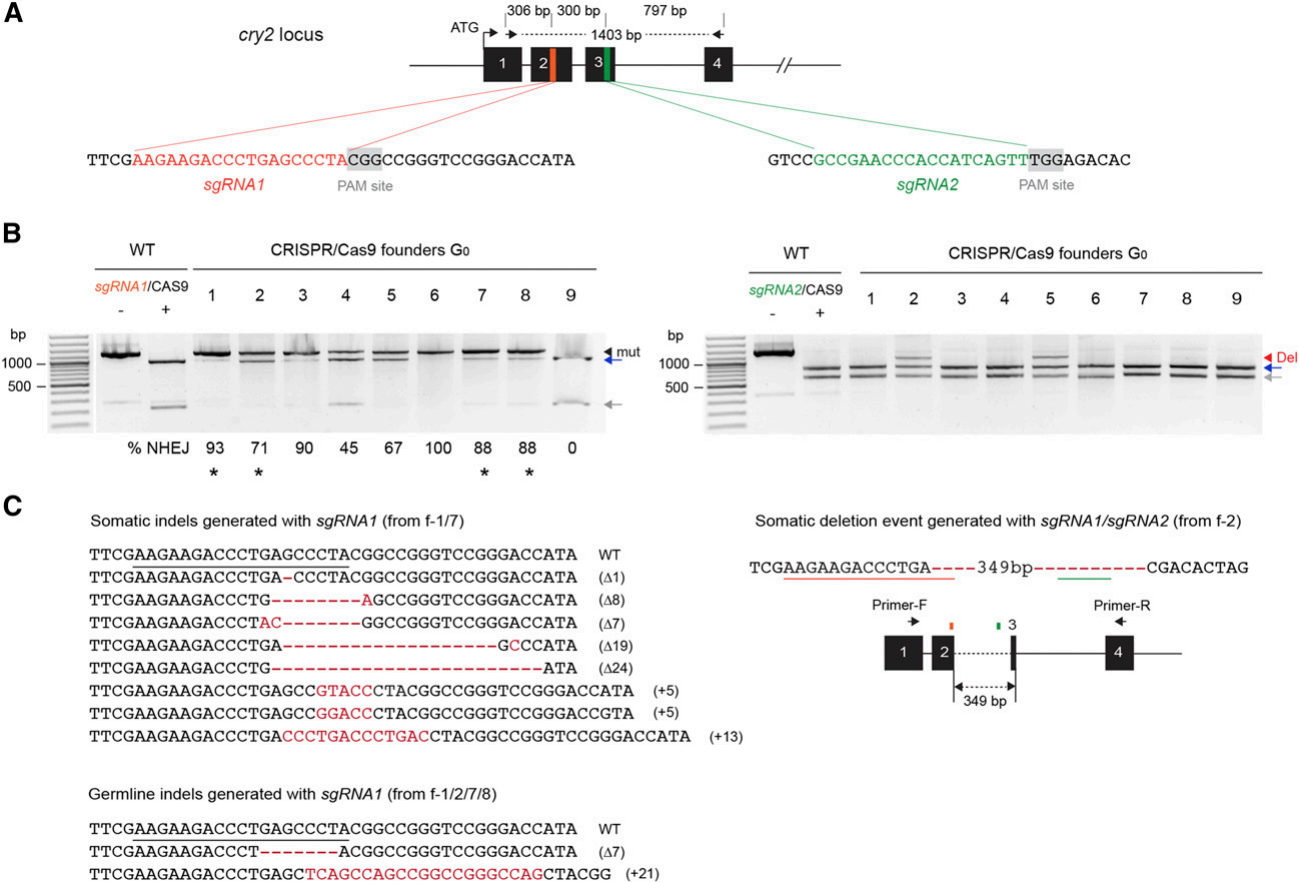
Highly efficient CRISPR/Cas9-mediated targeted mutations and deletions of monarch butterfly *cry2* using dual sgRNAs. (A) Schematic of part of the monarch *cry2* genomic locus containing the two CRISPR/Cas9 target sites. The orange and green regions in exons 2 and 3 are expanded to provide the sequences targeted for genome editing by each single guide RNA (sgRNA). Protospacer adjacent motifs (PAM site, 5′-NGG-3′) are highlighted in gray. Arrows on top represent the positions of the primers used to amplify the 1403 bp targeted region for analysis of mutagenic lesions in B (*cry2* F2, *cry2* R2 in Table S1). (B) Detection of mutagenic lesions at the *cry2* loci targeted by sgRNA1 (left) and sgRNA2 (right) in somatic cells of founder G_0_ butterflies (9 out of 13 are shown). For each founder, a PCR fragment was subjected to a Cas9-based *in vitro* cleavage assay with each of the sgRNAs. Black arrowhead, amplicons carrying mutations (mut). Estimation of the frequency of NHEJ-mediated indels is provided under each founder. Red arrowhead, amplicons carrying genomic deletions (del) at the sites targeted by the sgRNAs. Blue and gray arrows, cleaved wild-type (WT) PCR fragments. Black stars, founders selected for crosses to determine germline targeting rates. (C) Left, CRISPR/Cas9-induced mutations in somatic and germline cells with sgRNA1. Guide RNA binding site is underlined on the wild-type sequence. Red dashes and red letters, deletion and insertions, respectively. Right, Dual sgRNA-mediated genomic deletion in somatic cells. Positions of sgRNA1 and sgRNA2 are underlined on the sequence and represented by colored boxes on a schematic representation. CRISPR, clustered regularly interspaced short palindromic repeats; NHEJ, nonhomologous end-joining; PCR, polymerase chain reaction.

We next investigated whether CRISPR/Cas9 could be widely applicable for genome editing in the monarch butterfly by targeting an additional locus, *cry2* (Figure 4). While the use of a single sgRNA proved to be successful for generating small indels, inducing deletions larger than a few bp with the simultaneous use of two sgRNAs would ultimately facilitate the screening of mutagenic lesions solely based on PCR-based fragment size differences (Liu *et al.* 2014). We thus coinjected newly oviposited monarch eggs with 0.1 μg/μl of two *in vitro* transcribed sgRNAs respectively complementary to 19 bp DNA sequences in the second and third exons of *cry2*, along with 0.5 μg/μl of mRNA encoding Cas9 (Figure 4A). Out of 88 injected embryos, 21.6% larvae hatched and about 15% (13/88) developed into healthy larvae. Similar to our findings at the *clk* locus, these hatching and survival rates were comparable to those obtained with TALENs injections (Table 1), suggesting that both TALENs and CRISPR/Cas9 are substantially less toxic than ZFNs to the butterflies. To simultaneously screen for both the presence of small indels and larger deletions in the 13 surviving mosaic larvae, we performed *in vitro* Cas9-based cleavage assays on 1403 bp PCR fragments using either sgRNA (sgRNA 1 and sgRNA2; Figure 4B). However, only results with the sgRNA2 can inform for both the presence of small indels and larger deletions as a deletion resulting from DSBs between the two sgRNAs would result in a fragment of similar size than the cleaved wild-type product by sgRNA1 (∼1085 bp; Figure 4A). Nevertheless, we detected a surprisingly high frequency of targeted mutagenesis at the site targeted with sgRNA1 in 8 out of 13 founders G_0_, with rates ranging from 45–100% (Figure 4B, left panel) and lesions harboring a variety of small indels in somatic cells (Figure 4C).

At the site targeted with sgRNA2, we only detected mutagenic lesions in two of the 13 larvae genotyped. However, these legions were large deletions of ∼350 bp (Figure 4B, right panel; founders #2 and #5). Cloning of the corresponding PCR fragments confirmed that they resulted in a 349 bp deletion between the two target sites (Figure 4B and Figure S3). Although these data suggested that the DNA cleavage mediated by sgRNA2 was likely inefficient, they also suggested that when two DSBs were introduced, the occurrence of large deletions was likely favored. Testing whether these large deletions were transmitted to the germline was hindered by the fact the butterflies did not survive to reproductive maturity. However, we were able to provide evidence of germline transmitted indels generated with sgRNA1 in the two larvae that eclosed from two backcrossed founders, presenting the highest rates of somatic targeting (Figure 4C and Table 1). This low progeny number might be due to the fact that high targeting of monarch *cry2* negatively impacts egg production, as we previously observed that *cry2* knockout female monarchs develop few to no mature eggs (unpublished data).

### Targeted mutagenesis of clk disrupts the monarch circadian clockwork

**Figure 5:**
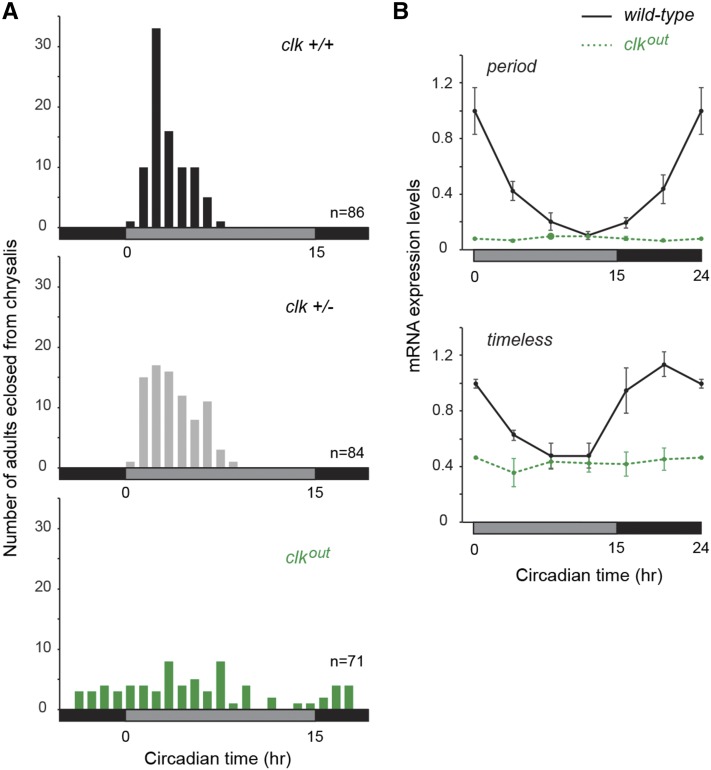
CLOCK deficiency disrupts circadian behavior and the molecular clockwork. (A) Profiles of adult eclosion in constant darkness (DD) of wild-type (^+/+^, black), heterozygous (^+/−^, gray) and homozygous mutant (*^out^*, green) siblings of a *clock (clk)* mutant line carrying a 4 bp deletion in exon 3, entrained to LD (15 hr light, 9 hr dark) throughout their larval and pupal stages. Eclosion occurred on the first and second d of DD. Data from both d are pooled and binned in 1 hr intervals. Effect of genotype on eclosion time, one-way ANOVA: *P* < 0.0002; Tukey posthoc test: *clk^+/+^* *vs.* *clk^+/−^*, *P* > 0.05; *clk^+/+^* *vs.* *clk^out^*, *P* < 0.01; *clk^+/−^* *vs.* *clk^out^*, *P* < 0.01. Black horizontal bars, subjective night; gray horizontal bars, subjective day. (B) Circadian expression of *period* and *timeless* in brains of wild-type (black lines) and homozygous mutant (dashed green lines) siblings of the *clk* mutant line entrained to LD throughout their larval and pupal stages. Values are mean ± SEM of three animals, except for circadian time 0 (CT0) in *clk*^*out*^, which represents the mean of two animals. Interaction genotype × time, two-way ANOVA: *per*, *P* < 0.0001; *tim*, *P* < 0.01. Box shading: gray, subjective day; black, subjective night.

The occurrence of a mammalian-like core component in the monarch (CRY2), and the fact that mouse CLK is not required for central circadian clock function (Debruyne *et al.* 2006), prompted us to characterize monarch CLK function *in vivo* to unambiguously determine whether it is essential for central clock function in the brain. To assess the effect of knocking out monarch CLK on circadian behavior, we examined the timing of butterfly pupal eclosion (*i.e.*, the emergence of the adult from its pupal case), a robust and easily tractable behavior under the control of the brain circadian clock (Froy *et al.* 2003; Merlin *et al.* 2013). We focused our analysis on female knockouts, because the location of *clk* on the Z chromosome permitted the recovery of female knockouts from every backcross of heterozygote males to wild-type females. This strategy allowed for a rapid behavioral analysis of *clk* female knockouts (*clk^out^*) generated through three independent and consecutive backcrosses, thereby reducing the chances that the phenotype observed result from potential off-target effects. We found that the circadian timing of adult eclosion was disrupted in *clk* knockouts, while sibling wild-type and heterozygote butterflies eclosed rhythmically, exhibiting a peak of eclosion in the early subjective day at the populational level (Figure 5A; one-way ANOVA: *P* < 0.0002; Tukey posthoc test: *clk^+/+^* *vs.* *clk^+/−^*, *P* > 0.05; *clk^+/+^* *vs.* *clk^out^*, *P* < 0.01; *clk^+/−^* *vs.* *clk^out^*, *P* < 0.01).

To determine how the molecular clock was affected by CLK deficiency, we quantified by quantitative real-time PCR the expression of two core clock components, *period* and *timeless*, from wild-type and *clk^out^* homozygous monarch brains, the location of circadian clocks controlling insect eclosion behavior (Zhu *et al.* 2008). While *period* and *timeless* robustly cycled in brains of wild-type butterflies, their rhythms were abolished in brains of *clk*^*out*^ and their expression levels were constitutively low (Figure 5B; two-way ANOVA, interaction genotype × time: *per*, *P* < 0.0001; *tim*, *P* < 0.01). These behavioral and molecular data confirm that monarch CLK is functioning as an essential component of the CLK:CYC transcriptional activator complex of the monarch circadian clock, similar to its *Drosophila* homolog (Allada *et al.* 1998; Mahesh *et al.* 2014).

## Discussion

The ability to harness efficient reverse-genetic approaches in butterflies is critical for these species to provide powerful model systems. Even though the use of artificial nucleases has proven to be successful for targeted mutagenesis in two butterfly species, the monarch butterfly and the swallowtail butterfly (Merlin *et al.* 2013; Li *et al.* 2015), the rapid adaptation of these technologies to other lepidopterans has been hindered by an absence of straightforward and practical strategies to introduce targeted mutations and identify carriers in particular for genes without morphological phenotypes. Here, we demonstrate that TALENs and CRISPR/Cas9 mediate highly efficient, heritable, targeted mutagenesis at two genomic loci in the monarch butterfly. Our study provides a simple workflow to recover germline mutants in the progeny of a small number of mosaic butterflies obtained from less than 100 injected eggs, and establish knockout lines in approximately 3 months with minimal injection and screening efforts. In the process of expanding the monarch genetic toolbox, we also generated a monarch knockout for *clk* and defined its critical function as encoding a transcriptional activator of the circadian clock used by monarchs during their migration, providing a valuable resource to further our understanding of the clock control of this spectacular behavior.

As previously reported, the use of TALENs and/or CRISPR/Cas9 for nonmodel insect genome editing confers several advantages over ZFNs: target selection is more flexible, their assembly is easier (Cermak *et al.* 2011; Jinek *et al.* 2012), and their success in attaining high mutagenesis efficiencies is greater (Ma *et al.* 2012; Watanabe *et al.* 2012; Sajwan *et al.* 2013; Wang *et al.* 2013; Liu *et al.* 2014; Wei *et al.* 2014; Gilles *et al.* 2015; Li *et al.* 2015). Our study in the monarch shows that both technologies substantially increase targeting efficiencies in somatic cells by almost an order of magnitude in comparison to ZFNs, with rates as high as 100% in the case of CRISPR/Cas9. However, the targeting efficiencies we observed appeared to be locus-dependent, with greater efficiencies of *cry2* targeting using CRISPR/Cas9 and *clk* targeting using TALENs. Whether these differences are due to different sgRNAs achieving different mutation rates, or to differences in local DNA accessibility, remains unclear.

In spite of the broad interest in the use of CRISPR/Cas9 for targeted mutagenesis in nonmodel insects, the general lack of simple and inexpensive assays to quantitatively assess rates of targeting in individual mutants with no visible phenotype has limited the generation of knockout lines to only a few species (Gratz *et al.* 2013; Ma *et al.* 2014; Wei *et al.* 2014; Awata *et al.* 2015; Basu *et al.* 2015; Dong *et al.* 2015; Gilles *et al.* 2015; Kistler *et al.* 2015). The quantitative methods that we used, which rely on Cas9-based *in vitro* cleavage assays (Kim *et al.* 2014), not only permit the rapid identification of highly targeted founders, but do so at low cost, allowing us to introduce an additional screening step into our workflow. Selecting only a few of the most promising candidates for crosses is sufficient to recover germline mutants, which avoids the burden associated with breeding a large number of individuals and screening their progeny for mutant alleles. Similar strategies could be implemented to accelerate the use of CRISPR/Cas9-mediated genome editing in other nonmodel insect species. Compared to TALENs, CRISPR/Cas9 can also be more readily adapted for multiplex gene editing to engineer large genomic deletions or knockout several genes simultaneously (Ma *et al.* 2014). While our study provides a proof-of-principle for CRISPR/Cas9-mediated multiplexing in the monarch butterfly, its success rate could be greatly enhanced by testing individually each sgRNA and selecting the most active ones in multiplex experiments. Importantly, because of its simplicity of use and efficacy, CRISPR/Cas9 offers the possibility of developing knock-in approaches in the monarch to tag protein coding genes and facilitate site-specific recombination and the creation of conditional alleles.

In conclusion, the methods we established for the rapid generation of heritable targeted mutations in the monarch butterfly should be widely applicable to other lepidopterans. Nuclease technology will also ultimately enable the systematic dissection of the monarch molecular clock mechanisms and the genetic basis of its long-distance migration. The threat to the North American monarch migration posed by ongoing climate change and habitat loss both across the major flyway in the US and at the overwintering sites in Mexico has prompted national conservation efforts to protect and restore their habitat. Understanding the genetic system underlying the monarch migratory program may help strengthen current conservation strategies.

## 

## Supplementary Material

Supporting Materials
