# Supplementary material for: Genomic Access to Monarch Migration Using TALEN and CRISPR/Cas9-Mediated Targeted Mutagenesis
Source: G3 (Bethesda). 2016 Feb 1;6(4):905–15. doi: 10.1534/g3.116.027029 (PMC4825660; doi:10.1534/g3.116.027029)
Supplement: Supporting Materials [file supp_6_4_905__index.html]

Genomic Access to Monarch Migration Using TALEN and CRISPR/Cas9-Mediated Targeted Mutagenesis — Supporting Materials 

# Genomic Access to Monarch Migration Using TALEN and CRISPR/Cas9-Mediated Targeted Mutagenesis

## Supporting Materials for Markert *et al.*, 2016

**Files in this Data Supplement:**

- Figure S1 - Targeted mutagenesis induced by microinjection of TALEN mRNAs into monarch butterfly embryos. (.pdf, 108 KB)
- Figure S2 - Circadian expression of *clock* in brains of wild-type monarchs.(.pdf, 37 KB)
- Figure S3 - Sequence of the genomic deletion generated using two sgRNAs at the *cry2* locus. (.pdf, 43 KB)
- Table S1 - Primer sequences. (.pdf, 124 KB)
