# Supplementary material for: Genomic Access to Monarch Migration Using TALEN and CRISPR/Cas9-Mediated Targeted Mutagenesis
Source: G3 (Bethesda). 2016 Feb 1;6(4):905–15. doi: 10.1534/g3.116.027029 (PMC4825660; doi:10.1534/g3.116.027029)
Supplement: Supporting Materials [file supp_g3.116.027029_FigureS1.pdf]

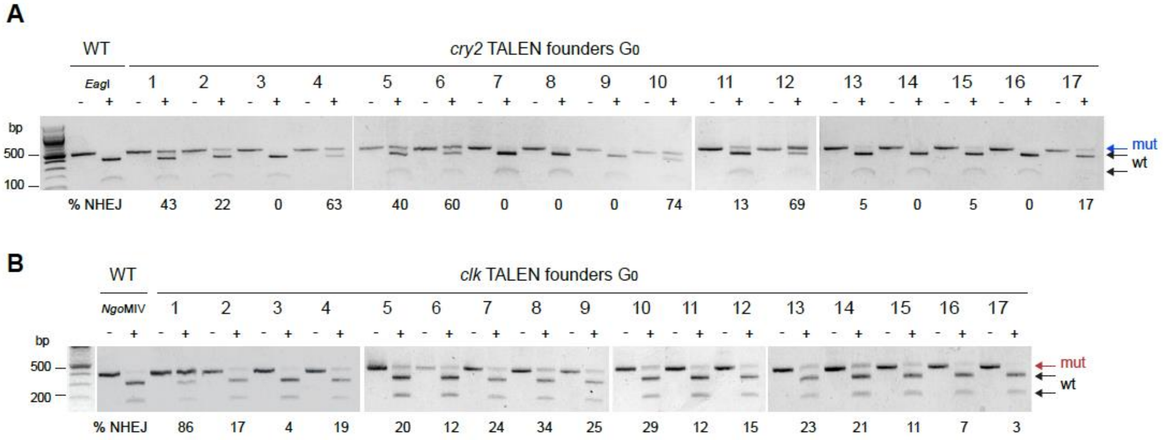

Figure S1. Targeted mutagenesis induced by microinjection of TALEN mRNAs into monarch butterfly embryos. (A) Targeting mutagenesis at the *cry2* locus for all alive founders validated by PCR and *Eag*I digestion. For each founder, the PCR product was either not subjected (-) or subjected to *Eag*I (+). The blue arrow represents genomic amplicons carrying targeted mutations that are resistant to *Eag*I digestion and the black arrows represent cleaved wild-type fragments. (B) Targeting mutagenesis at the *clock* locus for all alive founders validated by PCR and *Ngo*MIV digestion. For each founder, the PCR product was either not subjected (-) or subjected to *Ngo*MIV (+). The red arrow represents genomic amplicons carrying targeted mutations that are resistant to *Ngo*MIV digestion and the black arrows represent cleaved wild-type fragments. Estimation of the frequency of NHEJ-mediated indels is provided under each founder.
