# Supplementary material for: Genomic Access to Monarch Migration Using TALEN and CRISPR/Cas9-Mediated Targeted Mutagenesis
Source: G3 (Bethesda). 2016 Feb 1;6(4):905–15. doi: 10.1534/g3.116.027029 (PMC4825660; doi:10.1534/g3.116.027029)
Supplement: Supporting Materials [file supp_g3.116.027029_FigureS2.pdf]

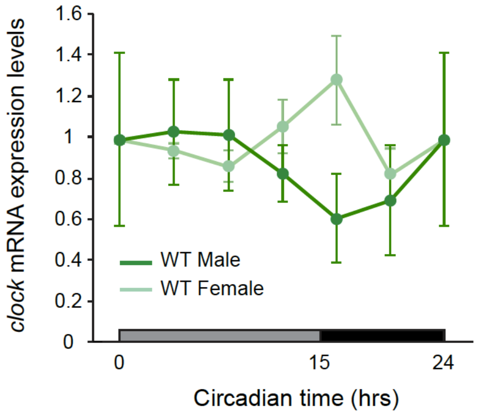

Figure S2. Circadian expression of *clock* in brains of wild-type monarchs.

Expression levels in brains of females (light green) and males (dark green) entrained to light:dark cycles, and collected the first day of transfer in constant darkness. Values are mean ± SEM of three animals. Box shading: gray, subjective day; black, subjective night. Effect of sex, two-way ANOVA: p=0.20.
