# Supplementary material for: Genomic Access to Monarch Migration Using TALEN and CRISPR/Cas9-Mediated Targeted Mutagenesis
Source: G3 (Bethesda). 2016 Feb 1;6(4):905–15. doi: 10.1534/g3.116.027029 (PMC4825660; doi:10.1534/g3.116.027029)
Supplement: Supporting Materials [file supp_g3.116.027029_FigureS3.pdf]

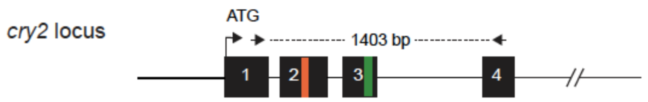

sgRNA1

```
WT         TTCGAAGAAGACCCTGAGCCCTACGGCCGGGTCCGGGACCATAATATTATGACCAAATGC 60
Del f-2    TTCGAAGAAGACCCTGAG---------CCGTGTACGG----------------------- 28
           ******************         *** **.***

WT         CGCGAAGTCGGTATTCAAGTAACTTCAAGAGTATCACACACCTTGTACAAATTAGATGAG 120
Del f-2    ------------------------------------------------------------


WT         TAAGTGTTACTGACGTGGTTTGTGTATCCGTGGGTTAAGTGAAATGCGACGATTACGTAA 180
Del f-2    -------TACGGGC---------------------------------------------- 35
                  *** *.*

WT         ATTTTTAATTGCGTATTGTTATTGTGTCGTGGTTTTAGTATAATTGAGAAGAATGGTGGT 240
Del f-2    ------------------------------------------------------------


WT         AAGGCGCCGCTGACCTATCATCAGTTCCAAGCGCTGATAGCGAGCATGCCCCCGCCTCCG 300
Del f-2    ------------------------------------------------------------
```

sgRNA2

```
WT         TCCGCCGAACCCACCATCAGTTTGGAGACACTCAACCGCGCCGTTACACCTATCTCGGAT 360
Del f-2    ------------------------------------------------------------


WT         AATCACGACGAACGATTTGGAGTGCCGACACTCGAAGAACTTGGTATTAATTTATAATTA 420
Del f-2    ------------------------ACGACACTAGAAGAACTTGGTATTAATTTATAACTA 71
                                   .*******.************************ **

WT         AAAGCTATGCCCTCGTTATATTTTTTCTCATCATATATTCAATGACAGTTTAATAGGTTA 480
Del f-2    AAAGCTATGCCCTCGTTATAATTTTTCTCATCATATATTCAATGACAGTTCAATAGGTTA 131
           ********************:***************************** *********

WT         GATAGCTTTTAAACTATCGTCCAATTAAAGCGACGTTTAAATGCAATAAATATTTTCTTT 540
Del f-2    GATAGCTTTTAAACTATCGTCCAATTAAAGCGACGTTTAAATGCAATAAATATTTTCTCT 191
           ********************************************************** *
```

Figure S3. Sequence of the genomic deletion generated using two sgRNAs at the *cry2* locus. The position of the two sgRNA in exons 2 and 3 is shown on the schematic of the locus. Wild-type and mutated sequences are aligned to show the nature of the deletion.
