# Supplementary material for: Genomic Access to Monarch Migration Using TALEN and CRISPR/Cas9-Mediated Targeted Mutagenesis
Source: G3 (Bethesda). 2016 Feb 1;6(4):905–15. doi: 10.1534/g3.116.027029 (PMC4825660; doi:10.1534/g3.116.027029)
Supplement: Supporting Materials [file supp_g3.116.027029_TableS1.pdf]

Table S1. Primer sequences.

Clock and *cry2* primers	
clock F1	GGAGAGACCAGTTCAACATGC
clock F2	GATGGGAATAAAGATACGGGTCAC
clock R1	CGGAGTATGCCCTAGCAAAG
clock R2	CCAAGGCCTCCAAGACTAAGTA
cry2 F1	ATTCCCGTTTATTCGTGGTG
cry2 F2	GAGAGAGGGGCTCGTTGATG
cry2 R1	TCGAGTGTCGGCACTCCAAATC
cry2 R2	CTGGCGTCATCTTCGGTCTT
